# Short-Term Diet Induced Changes in the Central and Circulating IGF Systems Are Sex Specific

**DOI:** 10.3389/fendo.2020.00513

**Published:** 2020-08-11

**Authors:** Santiago Guerra-Cantera, Laura M. Frago, Francisca Díaz, Purificacion Ros, Maria Jiménez-Hernaiz, Alejandra Freire-Regatillo, Vicente Barrios, Jesús Argente, Julie A. Chowen

**Affiliations:** ^1^Department of Endocrinology, Hospital Infantil Universitario Niño Jesús, Instituto de Investigación La Princesa, Madrid, Spain; ^2^Department of Pediatrics, Universidad Autónoma de Madrid, Madrid, Spain; ^3^Centro de Investigación Biomédica en Red de Fisiopatología de la Obesidad y Nutrición (CIBEROBN), Instituto de Salud Carlos III, Madrid, Spain; ^4^Hospital Universitario Puerta de Hierro-Majadahonda, Madrid, Spain; ^5^IMDEA Food Institute, CEI UAM + CSIC, Madrid, Spain

**Keywords:** obesity, IGFs, IGFBP2, PAPP-A, stanniocalcins, high fat diet, hypothalamus, sex differences

## Abstract

Insulin-like growth factor (IGF) 1 exerts a wide range of functions in mammalians participating not only in the control of growth and metabolism, but also in other actions such as neuroprotection. Nutritional status modifies the IGF system, although little is known regarding how diet affects the newest members of this system including pregnancy-associated plasma protein-A (PAPP-A) and PAPP-A2, proteases that liberate IGF from the IGF-binding proteins (IGFBPs), and stanniocalcins (STCs) that inhibit PAPP-A and PAPP-A2 activity. Here we explored if a 1-week dietary change to either a high-fat diet (HFD) or a low-fat diet (LFD) modifies the central and peripheral IGF systems in both male and female Wistar rats. The circulating IGF system showed sex differences in most of its members at baseline. Males had higher levels of both free (*p* < 0.001) and total IGF1 (*p* < 0.001), as well as IGFBP3 (*p* < 0.001), IGFBP5 (*p* < 0.001), and insulin (*p* < 0.01). In contrast, females had higher serum levels of PAPP-A2 (*p* < 0.05) and IGFBP2 (*p* < 0.001). The responses to a short-term dietary change were both diet and sex specific. Circulating levels of IGF2 increased in response to LFD intake in females (*p* < 0.001) and decreased in response to HFD intake in males (*p* < 0.001). In females, LFD intake also decreased circulating IGFBP2 levels (*p* < 0.001). In the hypothalamus LFD intake increased IGF2 (*p* < 0.01) and IGFBP2 mRNA (*p* < 0.001) levels, as well as the expression of NPY (*p* < 0.001) and AgRP (*p* < 0.01), but only in males. In conclusion, short-term LFD intake induced more changes in the peripheral and central IGF system than did short-term HFD intake. Moreover, these changes were sex-specific, with IGF2 and IGFBP2 being more highly affected than the other members of the IGF system. One of the main differences between the commercial LFD employed and the HFD or normal rodent chow is that the LFD has a significantly higher sucrose content, suggesting that this nutrient could be involved in the observed responses.

## Introduction

Insulin-like growth factor (IGF) 1 is involved in a wide range of functions (1, 2) including promotion of systemic growth through actions exerted directly on bone (3), anabolic effects promoting protein synthesis and glucose uptake in muscle (4) and stimulation of lipogenesis (5). Because of their structural similarity, IGF1 shares metabolic functions with insulin (6) and elevated levels of this growth factor reduce glycemia (7). In the brain IGF1 is involved in numerous functions including glucose metabolism (8), neural development (9), neural activity (10), synaptogenesis (11), adult neurogenesis (12), cognition (10), and amyloid clearance (13). It also exerts beneficial effects against inflammation (14) and neurodegeneration (9).

The main source of circulating IGF1 is the liver. However, there is also local production in most tissues including the brain, which is largely due to production by astrocytes and microglia (15, 16). The two ligands of the IGF system, IGF1 and IGF2, are secreted and bound to one of six different IGF-binding proteins (IGFBPs), thus modifying their biological activity. In addition to binding IGF1 or IGF2, IGFBP3, and IGFBP5 bind the acid labile subunit (ALS) to form a trimolecular complex of 150 KDa, which increases the half-life of the ligand (17). In the proximity of the target cell, proteases such as the metalloproteases pregnancy-associated plasma protein-A (PAPP-A) and PAPP-A2, cleave the junction between IGF1 or IGF2 with the IGFBP, allowing the free ligand to bind its receptor (18). Stanniocalcins (STCs) are a third level of regulation of this system, acting as endogenous inhibitors of the activity of both PAPP-A and PAPP-A2 and consequently reducing the release of both IGF1 and IGF2 (19, 20).

Nutritional status modifies circulating levels of IGF1, as well as of other members of the IGF system (21–24). High fat diet (HFD)-induced obesity has been shown to increase the expression of IGF2 in adipose tissue (25) and to inhibit the effects of IGF1 in chondrocytes (26), while IGF1 stimulates adipose tissue proliferation (5). Moreover, IGFBP2 is reported to participate in glucose metabolism and to be a target of leptin (27). The more recently described members of this family are also involved in metabolism as, for example, adult female PAPP-A knockout mice have been shown to be resistant to high fat/high sugar intake (28). High fat diet-induced and leptin-deficient obesity is associated with reduced STC2 synthesis in liver, with STC2 administration attenuating hyperlipidemia and steatosis (29). Recently, circulating levels of PAPP-A and PAPP-A2, as well as STC-2, were reported to be unchanged in response to metreleptin treatment in adult men and women (30). Thus, more information is required regarding the metabolic implications of the IGF system including the pappalysins and stanniocalcins.

Central IGF1 also modulates the neuroendocrine control of metabolism (31), but less is known regarding the participation of other members of this system at the central level in response to metabolic changes or to specific nutrients. Moreover, in recent years the important role of hypothalamic inflammation in obesity and its secondary complications has been obviated. As IGF1 exerts neuroprotective and anti-inflammatory effects (14), obesity or nutrition-induced modifications in this system could be involved in hypothalamic inflammation and central metabolic control.

In this study we aimed to determine the possible changes in the central and circulating IGF systems in response to short-term high-fat diet (HFD) or low-fat diet (LFD) consumption. Low fat diets have been commercialized as controls for the HFD, but their use as such is questionable and thus, we also compared the metabolic response between chow and LFD.

## Materials and Methods

### Ethical Statement

All experiments were designed according to the European Communities Council Directive (2010/63/UE) and the Royal Decree 53/2013 pertaining to the protection of experimental animals. This study was also approved by the Ethical Committee of Animal Experimentation of the Hospital Puerta de Hierro de Madrid and the Animal Welfare Organ of the Comunidad Autónoma de Madrid.

### Animals and Diets

Male and female postnatal day (PND) 50 Wistar rats were purchased from Charles River Laboratories and acclimated to the new environment for 13 days before dietary challenge. They were then randomly distributed between the three experimental groups for each sex (*n* = 6/group). Starting at PND 63, animals were fed *ad libitum* with either a HFD (62% kcal from fat, 18% kcal from proteins, 20% kcal from carbohydrates, 5.1 kcal/g, LabDiet), a LFD (10% kcal from fat, 18% kcal from proteins, 72% kcal from carbohydrates, 3.76 kcal/g, LabDiet) or standard rodent chow (6% kcal from fat, 17% kcal from proteins, 77% from carbohydrates, 3.41 kcal/g, Panlab) for 1 week (more information regarding the diets is shown in Table 1). The rats were given free access to tap water. Body weight was measured every 3 days until sacrifice on PND70. Food intake was determined throughout the study. Total kcals and the amount of energy contributed by fat were calculated. Energy efficiency was calculated as total weight gain (g) divided by total energy intake (kcal) during the week of study.

**Table 1:** Composition of the diets employed; normal rat chow, low fat diet (LFD), high fat diet (HFD).

	**Chow**	**LFD**	**HFD**
**ENERGY PROVIDED BY (kcal/g)**			
**Protein**	16.8	18	18.1
**Fat**	6.6	10.2	61.6
**Carbohydrates**	76.6	71.8	20.3
**INGREDIENTS (%)**			
**Protein**	14.3	16.9	23.1
**Fat**	2.5	4.3	34.9
Lard	–	1.9	31.66
Cholesterol (ppm)	–	18	301
Omega-3 FA	0.11	0.19	0.39
Saturated FA	0.52	1.14	13.68
Monounsaturated FA	0.53	1.3	14
Polyunsaturated FA	–	1.59	–
**Carbohydrates**	65.2	67.4	25.9
Sucrose	0.94	33.13	8.85
Starch/maltodextrin/dextrin	50.6	34.16	16.15
**Fiber**	3.7	4.7	6.5

### Tissues and Sacrifices

Twelve hours before sacrifice, animals were weighed and then fasted. The animals were killed between 09:00 and 11:00 by decapitation. Trunk blood was collected and after clotting it was centrifuged at 3,000 rpm for 15 min. The serum was aliquoted and stored at −80°C avoiding freeze-thaw cycles. Peripheral glucose levels were measured by using a Freestyle Optimum Neo glucometer (Abbott, Witney, UK).

After decapitation, hypothalami, defined rostrally by the optic chiasm and caudally by the anterior margin of the mammillary bodies, were dissected and then frozen at −80°C. The inguinal adipose depot (subcutaneous adipose tissue; SCAT) and the perigonadal adipose depot (visceral adipose tissue; VAT) were dissected and weighed. The amount of each adipose tissue depot is expressed as percentage of body weight [weight (mg) relative to body weight (g)].

### ELISA and Colorimetric Assays

Serum levels of free IGF1 (AnshLabs, Webster, Texas, USA), total IGF1 (Mediagnost, Reutlingen, Germany), IGF2 (BlueGene, Shangai, China), IGFBP2 (Mediagnost), IGFBP3 (Mediagnost), IGFBP5 (BlueGene), PAPP-A2 (BlueGene), insulin (Millipore, Burlington, Massachusetts, USA), and leptin (Millipore) were quantified by ELISA following the manufacturer's instructions. Non-esterified fatty-acids (NEFA) (Wako Diagnostics, Richmond, Virginia, USA) and triglycerides (Spin React, Girona, Spain) were measured by colorimetric assays as described by the manufacturers.

### Protein and RNA Extraction

Protein and RNA extraction from hypothalami was performed by using an RNeasy Plus Mini Kit (Qiagen, Hilden, Germany) according to the manufacturer's instructions. After tissue lysis and DNA elimination, 70% ethanol was mixed with the sample, and then placed in an RNeasy spin column and centrifuged. Protein was isolated from the same tissues by collecting the first elution from the RNeasy® Mini Spin columns. The eluted volume was mixed with 4 volumes of cold acetone and stored O/N at −20°C. Samples were centrifuged at 3,000 rpm at room temperature for 10 min and the acetone removed. The pellets were resuspended in a CHAPS hydrate (Sigma-Aldrich, Darmstadt, Germany) solution containing 7 M urea, 2 M thiourea, 4% CHAPS, 0.5% 1 M Tris pH 8.8, in distilled water and stored at −80°C until used. The Bradford method was employed for protein quantification by using Protein Assay Dye Reagent Concentrate (Bio-Rad Laboratories, Hercules, California, USA).

### Western Blotting

For Western blotting, 20–40 μg of protein, depending upon the target protein to be analyzed, were resolved in SDS-denaturing polyacrylamide gels. Proteins were transferred to previously activated PVDF membranes at 350 mA for 90 min.

Non-specific binding was blocked by incubating with 5% non-fat dried milk or bovine serum albumin (BSA, phosphorylated proteins) in TBS-T [Tris-buffered saline and 0.1% (v/v) Tween 20], which was also used for preparing the primary and secondary antibody solutions. The antibodies and their dilutions are shown in Table 2. Clarity Western ECL Substrate (Bio-Rad Laboratories) was employed to visualize the chemiluminiscent signal by ImageQuant Las 4000 Software (GE Healthcare Life Sciences, Barcelona, Spain). Each protein was normalized to actin levels, or total protein levels for phosphorylated proteins, in the same sample.

**Table 2:** Antibodies used for Western blotting.

**Antibody**	**Type**	**Dilution**	**Host**	**Commercial source**	**Reference**
Actin	Monoclonal	1:5,000	Mouse	NeoMarkers	1295-P1
AKT	Polyclonal	1:1,000	Goat	Santa Cruz	sc-1619
ERK	Monoclonal	1:1,000	Mouse	Santa Cruz	sc-135900
GAPDH	Polyclonal	1:4,000	Rabbit	Sigma-Aldrich	^#^G9545
GFAP	Monoclonal	1:3,000	Mouse	Sigma-Aldrich	G-3893
Iba1	Polyclonal	1:1,000	Rabbit	Synaptic Systems	234003
IRS1	Polyclonal	1:500	Rabbit	Millipore	^#^06-248
JNK	Monoclonal	1/1,000	Mouse	Santa Cruz	sc-1648
pAKT (Ser 473)	Polyclonal	1:1,000	Rabbit	Promega	G7441
pERK	Polyclonal	1:1,000	Rabbit	Cell Signaling	^#^9101
PI3K p110β	Polyclonal	1:1,000	Rabbit	Santa Cruz	sc-602
pIRS1 (Ser 789)	Polyclonal	1:750	Rabbit	Cell Signaling	^#^2389
pJNK	Polyclonal	1:3,000	Rabbit	Promega	V7932
α-goat HRP conjugated	Polyclonal	1:2,000	Rabbit	Thermo Fisher	^#^31402
α-mouse HRP conjugated	Polyclonal	1:2,000	Goat	Invitrogen	^#^31430
α-rabbit HRP conjugated	Polyclonal	1:2,000	Goat	Dako	P0448

*AKT, protein kinase B; ERK, extracellular signal-regulated kinases; GAPDH, Glyceraldehyde 3-phosphate dehydrogenase; GFAP, Glial fibrillary acidic protein; Iba1, Ionized calcium binding adaptor molecule 1; IRS1, insulin receptor substrate 1; PI3K, phosphatidylinositol 3-kinases; JNK, c-Jun N-terminal kinases; HRP, horseradish peroxidase*.

### Real Time qPCR

For RT-PCR, 0.5–1 μg of RNA was retro-transcribed to cDNA by using a High-capacity cDNA reverse transcriptase kit (Applied Biosystems, Carlsbad, California, USA) or NZY First-Strand cDNA Synthesis Kit (NZY Tech, Lisbon, Portugal). TaqMan probes (Table 3) were used for RT-PCR in an ABI PRISM 7000 or QuantStudio 3 Real-Time PCR System (both from Applied Biosystems). Phosphoglycerate kinase 1 (Pgk1) and 18S (Rps18) were used as endogenous housekeeping controls.

**Table 3:** List of Taqman probes used for RT-PCR.

**Gene**	**Reference**
Agouti-related peptide (*Agrp*)	Rn01431703_g1
Cocaine- and amphetamine-regulated transcript (*Cart*)	Rn00567382_m1
Insulin-like growth factor 1 (*Igf1*)	Rn99999087_m1
Insulin-like growth factor 1 receptor (*Igf1r*)	Rn01477918_m1
Insulin-like growth factor 2 (*Igf2*)	Rn01454518_m1
Insulin-like growth factor 2 receptor (*Igf2r*)	Rn01636937_m1
Insulin-like growth factor-binding protein 1 (*Igfbp1*)	Rn00565713_m1
Insulin-like growth factor-binding protein 2 (*Igfbp2*)	Rn00565473_m1
Insulin-like growth factor-binding protein 3 *(Igfbp3*)	Rn00561416_m1
Insulin-like growth factor-binding protein 4 (*Igfbp4*)	Rn01464112_m1
Insulin-like growth factor-binding protein 5 (*Igfbp5*)	Rn00563116_m1
Neuropeptide Y (*Npy*)	Rn01410145_m1
Pregnancy-associated plasma protein A (*Pappa*)	Rn01458295_m1
Phosphoglycerate kinase 1 (*Pgk1*)	Rn00821429_g1
Pro-opiomelanocortin (*Pomc*)	Rn00595020_m1
18S *(Rps18)*	Rn01428915_g1
Stanniocalcin 1 (*Stc1*)	Rn00579636_m1
Stanniocalcin 2 (*Stc2*)	Rn00573702_m1

For the mathematical analysis, the ΔΔCT method was employed with expression of the housekeeping gene used as the endogenous control. Relative levels of expression were determined by normalizing the results to levels in the male chow group.

### Statistical Analysis

Data are presented as mean ± SEM. Statistics was performed using SPSS 15.0 software. Two-way ANOVA with Bonferroni as the *post-hoc* test was used in each case, with sex and diet used as factors. Pearson correlation coefficient was also calculated to assess the linear correlation between variables. Values of *p* < 0.05 were considered significant.

## Results

### Body Composition

There was an effect of sex [*F*_(1, 35)_ = 575.9, *p* < 0.001], with males weighing more than females regardless of diet. Short-term HFD intake induced body weight gain, but exclusively in males [*F*_(2, 17)_ = 4.9, *p* < 0.05; Table 4].

**Table 4:** Effects of 1 week on a high fat diet (HFD), low fat diet (LFD), or normal rat chow on body composition, glycemia, serum levels of insulin, Homeostatic Model Assessment for Insulin Resistance (HOMA-IR), leptin, non-esterified fatty-acids (NEFA) and triglycerides, and energy intake in male and female rats.

	**Chow males**	**HFD males**	**LFD males**	**Chow females**	**HFD females**	**LFD females**	**Sig**.
Body weight (g)	348.3 ± 12.0	359.5 ± 9.9	340.5 ± 2.7	186.7 ± 6.2	202.2 ± 6.1	199.0 ± 6.5	a, *p* < 0.001
Weight gain (% baseline)	7.8 ± 0.5	9.5 ± 0.3^#^	8.2 ± 0.3	4.2 ± 0.6	6.3 ± 1.0	7.7 ± 1.4	a, *p* < 0.01
Total kcal/rat	482.5 ± 31.3	621.4 ± 80.9	556.5 ± 10.7	328.5 ± 17.5	398.7 ± 27.2	394.8 ± 20.8	a, *p* < 0.001 b, *p* = 0.05
Kcal/rat/day/100 g body weight	19.4 ± 1.9	23.9 ± 2.7	22.5 ± 0.4	24.1 ± 0.2	27.1 ± 0.7	27.2 ± 0.3	a, *p* < 0.01 b, *p* < 0.05
Kcal from fat (total)	28.5 ± 1.8	378.4 ± 49.5^#^	56.1 ± 0.4	20.1 ± 0.9^@^	256.2 ± 7.3^#^	41.1 ± 1.8^#^^;^^@^	*p* < 0.001
Energy efficiency (%)	5.5 ± 0.3	5.4 ± 0.8	4.9 ± 0.3	2.5 ± 0.1	3.0 ± 0.2	3.7 ± 0.4	a, *p* < 0.001
Visceral adipose tissue (%)	1.42 ± 0.18	1.74 ± 0.31	1.57 ± 0.08	0.70 ± 0.02	1.03 ± 0.14	0.96 ± 0.13	a, *p* < 0.01
Subcutaneous adipose tissue (%)	0.89 ± 0.10	1.01 ± 0.10	0.96 ± 0.09	0.62 ± 0.05	0.80 ± 0.12	0.74 ± 0.06	a, *p* < 0.01
Glycemia (mg/dl)	77.5 ± 3.4	74.7 ± 3.4	74.8 ± 3.9	78.5 ± 5.3	67.0 ± 3.0	76.0 ± 2.2	ns
Insulin (ng/ml)	4.25 ± 0.75	3.33 ± 0.33	3.09 ± 0.37	2.28 ± 0.25	2.14 ± 0.11	2.04 ± 0.22	a, *p* < 0.001
HOMA-IR	23.2 ± 4.5	14.7 ± 2.1	17.6 ± 2.9	13.1 ± 2.1	10.3 ± 0.9	11.1 ± 0.4	a, *p* < 0.01
Leptin (ng/ml)	3.25 ± 0.79	5.91 ± 1.14	3.99 ± 0.75	1.37 ± 0.11	1.88 ± 0.45	1.80 ± 0.46	a, *p* < 0.001
NEFA (mmol/l)	0.95 ± 0.09	1.04 ± 0.13	1.19 ± 0.12	1.00 ± 0.09	0.94 ± 0.06	1.19 ± 0.08	ns
Triglycerides (mg/dl)	74.4 ± 15.3	53.8 ± 4.9	69.4 ± 12.8	44.1 ± 9.4	20.0 ± 1.7	35.8 ± 9.2	a, *p* < 0.001

*a, overall sex effect; b, overall diet effect*;

# *different compared to chow rats of the same sex*;

@ *different between sexes on the same diet. ns, non-significant. n = 6, except for energy intake where n = 3*.

The amount of VAT [*F*_(1, 35)_ = 24.1, *p* < 0.01] and SCAT [*F*_(1, 35)_ = 10.2, *p* < 0.01] were affected by sex, with males having a greater percentage of adipose tissue in both depots compared to females. No dietary influence was seen on either of these parameters.

An effect of sex was observed on the number of kcal consumed per rat [*F*_(1, 17)_ = 31.8, *p* < 0.001; Table 4], with males consuming more energy than females regardless of diet. In addition, diet had an overall effect on this parameter [*F*_(2, 17)_ = 3.7, *p* = 0.05]. When caloric intake was adjusted according to body weight there continued to be a sex effect [*F*_(1, 17)_ = 13.3, *p* < 0.01], but in this case females had a higher energy intake. There was also an effect of diet [*F*_(2, 17)_ = 4.0, *p* < 0.05]. Energy efficiency, expressed as grams of weight gained per kcal consumed, was sex dependent [*F*_(1, 17)_ = 44.1, *p* < 0.001] with males having a higher index of energy efficiency than females.

Kilocalories from fat were affected both by sex [*F*_(1, 17)_ = 8.4, *p* < 0.05] and diet [*F*_(2, 17)_ = 126.5, *p* < 0.001], with an interaction between these factors [*F*_(2, 17)_ = 4.9, *p* < 0.05]. As expected, HFD rats consumed more kcal from fat compared to chow and LFD animals (*p* < 0.001). Moreover, fat consumption on the LFD was also higher than on the chow diet in both sexes (*p* < 0.001).

### Serum Levels of Metabolic Factors and IGF Family Members

There was an overall effect of sex on serum leptin [*F*_(1, 35)_ = 22.4, *p* < 0.001], insulin [*F*_(1, 34)_ = 18.6, *p* < 0.001], and triglyceride [*F*_(1, 35)_ = 15.9, *p* < 0.001] levels, as well as HOMA-IR index [*F*_(1, 33)_ = 15.3, *p* < 0.01], with males having overall higher values than females in all cases (Table 4). There was no effect of either sex or diet on glycemia or non-esterified fatty acids (NEFA) levels (Table 4).

**Figure 1:**
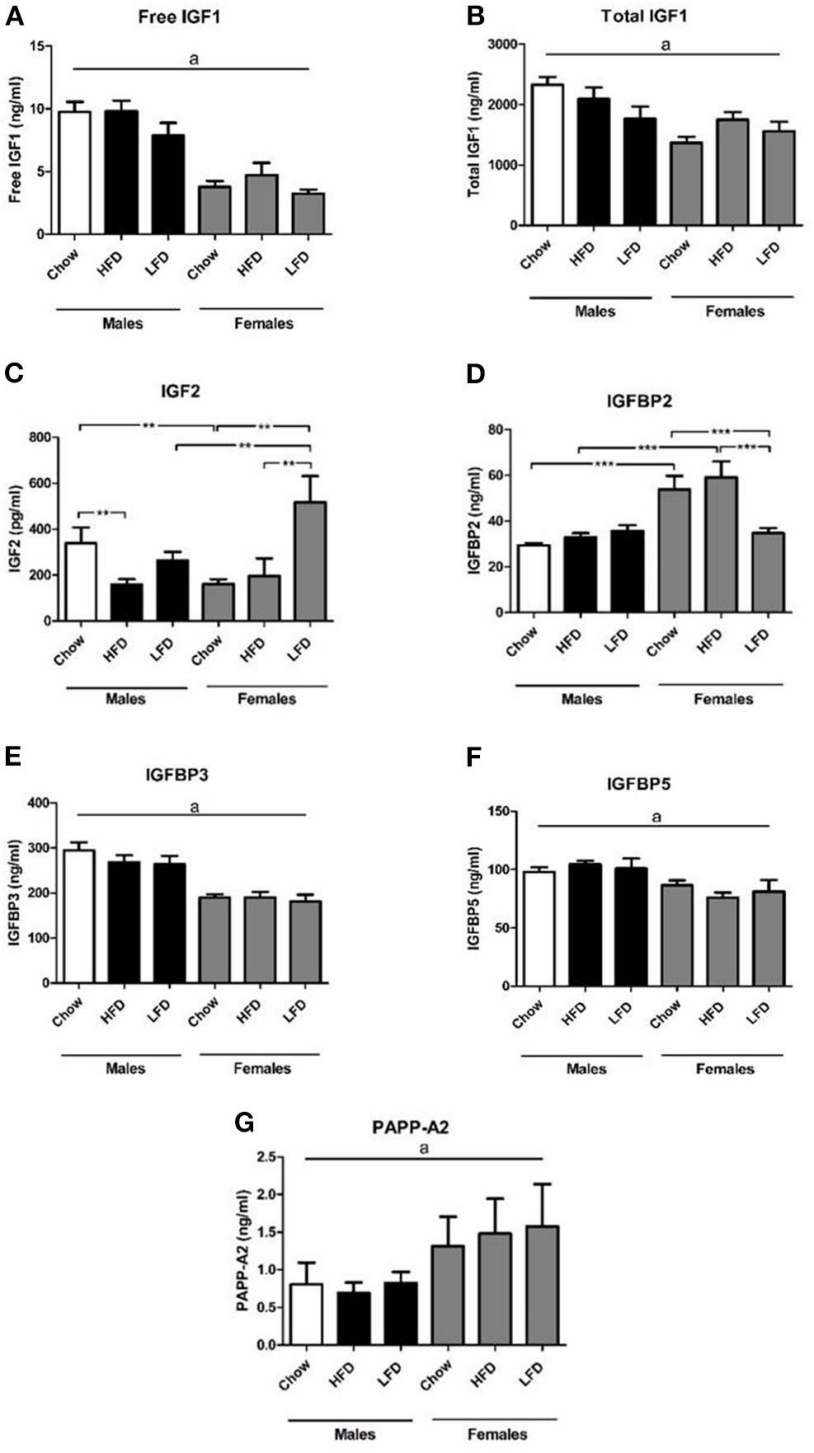
Serum levels of free insulin-like growth factor (IGF)-1 **(A)**, total IGF-1 **(B)**, IGF 2 **(C)**, IGF binding protien (IGFBP) 2 **(D)**, IGFBP3 **(E)**, IGFBP5 **(F)**, and pregnancy-associated plasma protein (PAPP-A)2 **(G)** in rats on a high fat diet (HFD), low fat diet (LFD) or a chow diet for 1 week. **p < 0.01; ***p < 0.001. a, overall effect of sex. *n* = 6.

Males had overall higher serum levels of free IGF1 [*F*_(1, 35)_ = 67.6, *p* < 0.001; Figure 1A] and total IGF1 [*F*_(1, 35)_ = 15.7, *p* < 0.001; Figure 1B] than females, with no dietary effect found. On a chow diet males had higher levels of IGF2 than females on the same diet [*F*_(1, 9)_ = 6.2, *p* < 0.05; Figure 1C]. In males, IGF2 levels were lower on the HFD compared to chow [*F*_(2, 15)_ = 3.6, *p* < 0.05]. In contrast, in females IGF2 levels were increased after LFD consumption compared to both chow and HFD [*F*_(2, 14)_ = 5.9, *p* < 0.05], with this resulting in females having higher levels than males when on the LFD [*F*_(1, 10)_ = 5.2, *p* < 0.05].

There was an effect of diet [*F*_(2, 35)_ =3.6, *p* < 0.05; Figure 1D] and sex [*F*_(1, 35)_ = 24.9, *p* < 0.001] on serum IGFBP2 levels, as well as an interaction between sex and diet [*F*_(2, 35)_ = 7.0, *p* < 0.01]. Females had higher levels than males when on chow [*F*_(1, 11)_ = 16.6, *p* < 0.01] or a HFD [*F*_(1, 11)_ = 13.4, *p* < 0.01]. In females, serum IGFBP2 levels were decreased after LFD consumption compared to both chow and HFD [*F*_(2, 17)_ = 5.6, *p* < 0.05].

Males had overall higher circulating IGFBP3 [*F*_(1, 35)_ = 53.2, *p* < 0.001; Figure 1E] and IGFBP5 [*F*_(1, 35)_ = 15.4, *p* < 0.001; Figure 1F] levels than females, with no dietary effect found. On the contrary, females had higher PAPP-A2 levels [*F*_(1, 30)_ = 4.3, *p* < 0.05; Figure 1G] in serum compared to males.

### Hypothalamic Response to Dietary Changes

**Figure 2:**
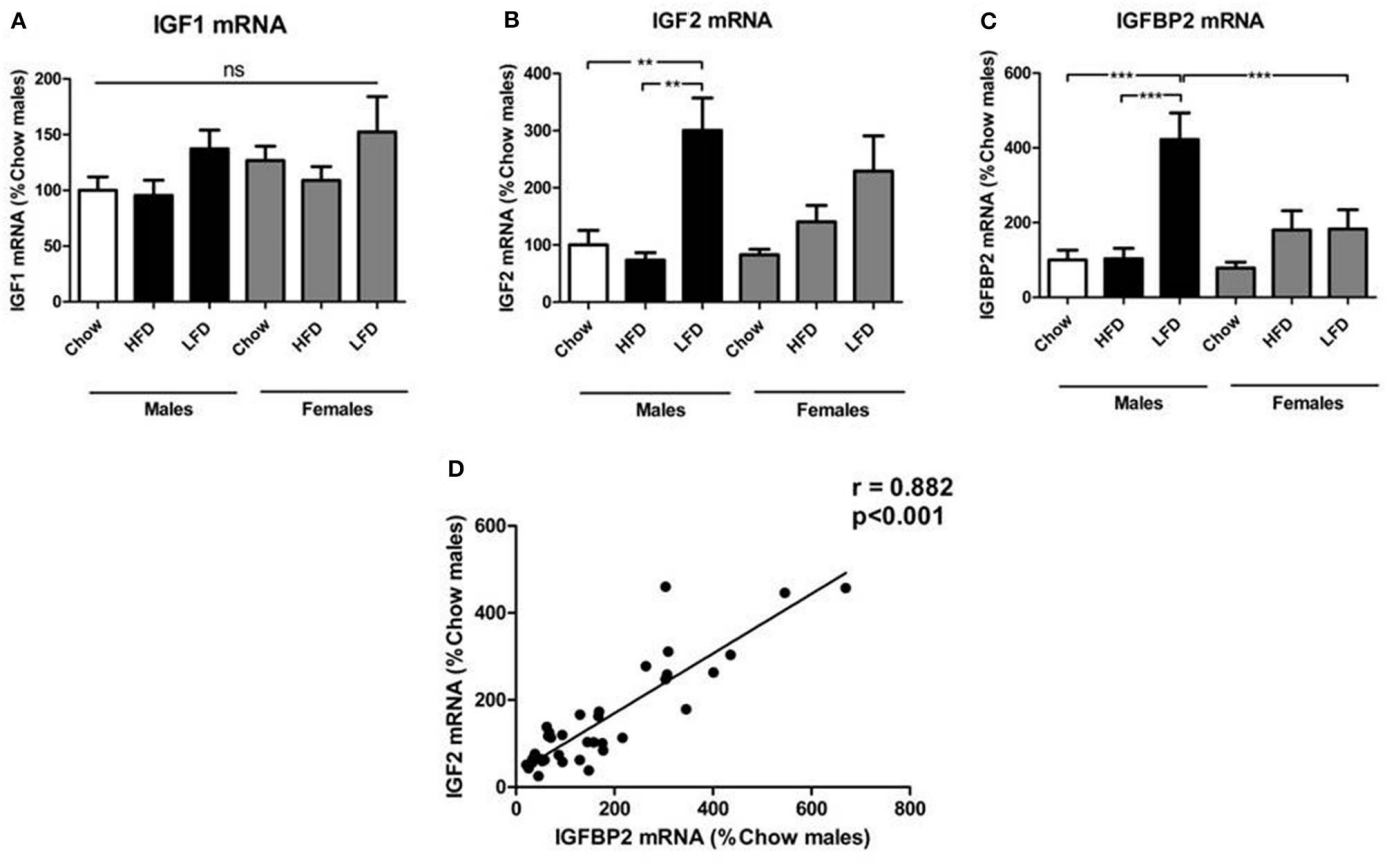
Relative mRNA levels of the insulin-like growth factor (IGF) system in the hypothalamus: IGF1 **(A)**, IGF2 **(B)**, IGF binding protien (IGFBP)2 **(C)**, and the correlation of relative hypothalamic mRNA levels of IGF2 and IGFBP2 **(D)**. **p < 0.01; ***p < 0.001; ns, non-significant; HFD, high fat diet; LFD, low fat diet. *n* = 6.

We found no effect of sex or diet on hypothalamic IGF1 mRNA levels (Figure 2A). Hypothalamic IGF2 mRNA levels were affected by diet [*F*_(2, 35)_ = 12.6, *p* < 0.01; Figure 2B], with an increase in response to LFD, reaching significance in males. Diet also affected IGFBP2 mRNA levels [*F*_(2, 35)_ = 12.4, *p* < 0.001; Figure 2C], which were increased by LFD intake with this being significant in males. Despite no sex differences being observed on a chow diet, relative expression of IGFBP2 in response to LFD was higher in males than females (*p* < 0.05). There was a positive correlation between the relative levels of hypothalamic IGF2 and IGFBP2 mRNA (r = 0.882, *p* < 0.001; Figure 2D).

There were no differences between groups in the relative hypothalamic mRNA levels of IGF-1R, IGF-2R, IGFBP1, IGFBP3, IGFBP4, IGFBP5, PAPP-A, STC-1, or STC-2 (Table 5).

**Table 5:** Relative gene expression of members of the IGF system in the hypothalamus.

	**Chow males**	**HFD males**	**LFD males**	**Chow females**	**HFD females**	**LFD females**	**Sig**.
IGF-1R	100.0 ± 12.2	106.2 ± 11.3	113.8 ± 10.0	110.6 ± 15.9	99.2 ± 9.0	133.1 ± 23.7	ns
IGF-2R	100.0 ± 14.3	95.5 ± 17.2	116.6 ± 12.6	99.6 ± 8.1	89.7 ± 16.0	109.2 ± 17.8	ns
IGFBP1	100.0 ± 10.5	122.3 ± 24.9	132.7 ± 17.1	126.5 ± 14.3	105.9 ± 15.9	120.7 ± 16.5	ns
IGFBP3	100.0 ± 13.2	97.7 ± 11.8	120.4 ± 14.9	105.7 ± 9.2	117.5 ± 14.3	128.5 ± 14.9	ns
IGFBP4	100.0 ± 23.7	91.4 ± 24.2	145.8 ± 25.1	94.7 ± 27.0	91.1 ± 13.8	119.0 ± 24.5	ns
IGFBP5	100.0 ± 19.4	91.6 ± 13.0	128.2 ± 16.7	86.2 ± 7.2	89.9 ± 12.6	121.9 ± 26.3	ns
PAPP-A	100.0 ± 6.5	104.4 ± 20.4	125.7 ± 21.1	109.8 ± 17.9	120.7 ± 22.7	116.8 ± 15.9	ns
STC-1	100.0 ± 12.2	101.3 ± 12.0	123.1 ± 21.5	121.6 ± 12.0	94.5 ± 13.4	115.2 ± 17.7	ns
STC-2	100.0 ± 18.2	105.8 ± 11.4	129.3 ± 15.3	98.9 ± 9.4	98.0 ± 11.5	121.3 ± 19.4	ns

*Data are represented as mean ± SEM. ns, non-significant. HFD, high fat diet; LFD, low fat diet. n = 6*.

**Figure 3:**
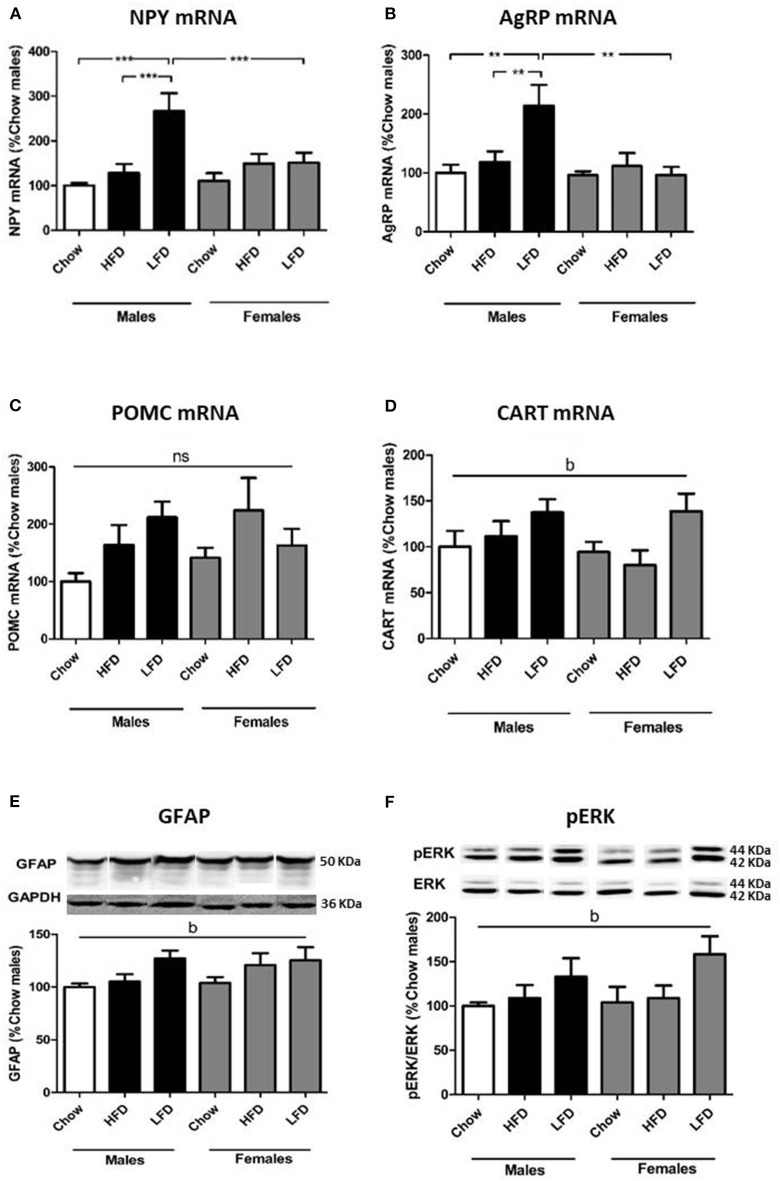
Relative mRNA levels of neuropeptide Y (NPY; **A**), Agouti related protein (AgRP; **B**), proopiomelanocortin (POMC; **C**) and cocaine and amphetamine regulated transcript (CART; **D**) and protein levels of glial fibrillary acidic protein (GFAP; **E**) and phophorylated extracellular signal-regulated kinase (pERK; **F**) in the hypothalamus of rats on a (HFD), low fat diet (LFD) or a chow diet for 1 week. These images are all from the same blot, but were not contiguous and for this reason they are individualy placed in order of the experimental groups in the graph. **p < 0.01; ***p < 0.001; b, effect of diet, ns, non-significant. *n* = 6.

The relative mRNA levels of neuropeptide Y [NPY; *F*_(2, 34)_ = 9.6, *p* < 0.01; Figure 3A] and Agouti-related protein [AgRP; *F*_(2, 34)_ = 3.9, *p* < 0.01; Figure 3B] were affected by diet, with an interaction between sex and diet [NPY: *F*_(2, 34)_ = 5.1, *p* < 0.05; AgRP: *F*_(2, 34)_ = 4.9, *p* < 0.05]. These orexigenic neuropeptides increased in response to LFD, but only in males [NPY: *F*_(2, 16)_ = 10.4, *p* < 0.01; AgRP: *F*_(2, 16)_ = 5.8, *p* < 0.05]. On a LFD, males had higher levels of both NPY [*F*_(1, 11)_ = 6.4, *p* < 0.05] and AgRP [*F*_(1, 11)_ = 9.5, *p* < 0.05] compared to females.

Proopiomelanocortin (POMC) mRNA levels showed no significant changes (Figure 3C). There was an overall dietary effect on cocaine and amphetamine-regulated transcript (CART) mRNA levels [*F*_(2, 34)_ = 4.7, *p* < 0.05; Figure 3D], with levels increasing in animals of both sexes on a LFD.

To assess if gliosis and hypothalamic inflammation were present, we analyzed glial fibrillary acidic protein (GFAP), ionized calcium-binding adapter molecule 1 (Iba1) and c-jun N-terminal kinase (JNK) activation. There was effect of diet on GFAP levels [*F*_(2, 35)_ = 4.1, *p* < 0.05; Figure 3E], with an increase in animals after LFD consumption. There was no effect on Iba1 or pJNK levels (Table 6).

To determine whether activation of the insulin/IGF signaling pathways in the hypothalamus was altered pIRS1, PI3K, pAKT (Ser473), and pERK were analyzed. There was an effect of diet on pERK levels [*F*_(2, 34)_ = 3.9, *p* < 0.05; Figure 3F], with an overall increase in animals on a LFD. No differences in pIRS1, PI3K, or pAKT were observed (Table 6).

**Table 6:** Effects of 1 week on a high fat diet (HFD), low fat diet (LFD) or chow diet on the phosphorylation of proteins involved in insulin and IGF signaling in the hypothalamus of male and female rats (*n* =6), as well as, Iba1: ionized calcium binding adaptor molecule 1 (Iba1), a marker of microglia and cell stress markers (JNK: c-Jun N-terminal kinases).

	**Chow males**	**HFD males**	**LFD males**	**Chow females**	**HFD females**	**LFD females**	**Sig**.
Iba1	100.0 ± 10.9	88.6 ± 12.0	97.1 ± 15.5	93.0 ± 14.1	90.9 ± 15.6	95.1 ± 18.5	n.s.
pAKT	100.0 ± 9.0	100.4 ± 8.3	103.6 ± 13.7	91.6 ± 11.1	87.8 ± 12.8	100.9 ± 20.6	n.s.
PI3KI	100.0 ± 13.1	151.9 ± 18.0	140.1 ± 14.3	155.3 ± 23.8	142.3 ± 15.1	119.1 ± 19.9	n.s.
pIRS1	100.0 ± 22.1	148.3 ± 21.8	183.0 ± 30.0	152.4 ± 31.2	132.4 ± 33.3	196.7 ± 55.7	n.s.
pJNK	100.0 ± 1.1	104.9 ± 3.0	104.9 ± 5.9	101.9 ± 2.7	99.0 ± 1.8	98.6 ± 4.5	n.s.

*Phosphorylated proteins were normalized with the total form of the protein. ns, non-significant*.

## Discussion

Here we show that there were not only sex differences in the IGF system's response to a short-term dietary change, but baseline serum levels of all members of the IGF system studied were significantly different between male and female rats. We observed higher levels of free IGF1, total IGF1, IGFBP3, and IGFBP5, as well as insulin in males compared to females. The GH secretory pattern differs between male and female rodents (32, 33) and could underlie some of these sex differences in the IGF system. Males have been reported to have higher IGF1 and IGFBP3 levels than females (34, 35), as found here. However, Frystyk and colleagues reported no sex differences in free IGF1 levels. It is possible that the employment of different methodologies to determine free IGF1 levels underlies this discrepancy in results. Although males were found to have higher circulating levels of most members of the IGF system, females had higher circulating levels of PAPP-A2, which could promote the availability of IGF1 for the tissues. The sex differences in the GH-IGF system are at least in part due to differences in sex steroid levels both during development and adulthood (36–38) and underlie the differences between males and females in growth and body size. More recently, the IGF system has also been implicated in the sex differences in the response to or propensity to develop different pathologies, as well as longevity (39).

Baseline sex differences in body weight and metabolism were observed, with males having a greater body weight, weight gain, energy intake, energy efficiency, and circulating leptin levels compared to females, as previously reported (40–42), as well as higher serum triglycerides levels. After 1 week on a HFD we found few effects on body weight or body composition, which is in accordance with some previous studies in rodents (42, 43), but not others (44). The LFD group was included in this study as this diet has been widely used as a control group in diet-induced obesity (DIO) models (26, 45, 46), but the higher content of carbohydrates, which is largely composed of sucrose, compared to HFD may also have metabolic effects. Neither diet affected final body weight in either sex, although the percentage weight gain in males on the HFD was significantly greater compared to the other groups. This is in accordance with their higher energy intake, as well as greater intake of kcals from fat compared to the other groups. Circulating levels of leptin are directly correlated with the amount of adipose tissue (47) and neither serum leptin levels nor adipose content were modified here on this short-term HFD. No changes in circulating levels of triglycerides, insulin or leptin levels were seen on the short-term HFD, as reported by others in mice (48).

The circulating IGF system is modified in human subjects with obesity. Serum levels of free IGF1 and IGFBP3, but not of total IGF1, are reported to be higher and IGFBP2 lower (22, 49) in children with obesity compared to control children. Serum IGF1 levels have also been reported to be higher when visceral adipose tissue content is elevated although a direct correlation of circulating IGF levels and BMI was not observed (50), suggesting the possible relevance of adipose distribution. In the study reported here, the lack of changes in IGF1 and IGFBP3 is probably due to the limited time of exposure to this diet and the lack of adipose accumulation. Indeed, the observed changes in the IGF system in obese subjects are most likely explained by their overall metabolic status rather than a direct response to specific nutrients or a specific diet as studied here.

Circulating IGF2 levels were affected by the short-term dietary changes, even though there was no significant modification in body weight; moreover, these changes were different between the sexes. In males, circulating IGF2 levels were reduced during HFD consumption, with HFD having no effect on this parameter in females. The response of this growth factor to metabolic changes is less clear with circulating IGF2 levels reported to being both reduced (51) and increased (52, 53) in obese compared to non-obese men and women. Shandu et al. found that serum IGF2 levels were reduced in subjects who gained weight during the study compared to those who maintained or lost weight and that lower serum IGF2 levels are negatively correlated with the risk to gain weight (51). Thus, the observed reduction in circulating IGF2 levels in males on the HFD could be a predictor of potential weight gain and metabolic risk. This reduction in IGF2 was not observed in females and this could be related to the observation that young adult female rodents tend be more resistant to HFD-induced weight gain (54, 55), which is in concordance with this growth factor possibly being an indicator of early metabolic changes.

Circulating levels of IGF2 were also affected by LFD intake and this also occurred in a sex specific manner. Rats of both sexes given the LFD had a higher energy intake compared to those on the chow diet, which is possibly due to the novelty and/or palatability of the diet and thus increased consumption, although increased energy intake of LFD compared to chow over a longer time-period has also been reported in male C57 mice (56) suggesting that this increase is not due only to novelty. In contrast to the HFD, LFD increased circulating levels of IGF2 and this effect was only observed in females. IGF2 participates in bone growth, adipose tissue accumulation and glucose metabolism, stimulating glucose uptake by adipocytes and acting directly at the level of the pancreas (57–59). Thus, it is possible that the high sucrose content of the LFD is involved in stimulating this rise in IGFBP2. LFD intake also decreased serum IGFBP2 levels, and again this effect of LFD was only found in females. Plasma IGFBP2 levels are reported to be negatively correlated with BMI (49, 60), as well as with adipogenesis and lipogenesis (61). IGFBP2 is the second most abundant IGF-binding protein in the circulation (62, 63) and has been suggested to be protective against obesity and to improve glucose tolerance on a HFD (27, 64). IGFBP2 binds IGF2 with a slight preference over IGF1 [reviewed by (65)] and the decrease in IGFBP2 in LFD females may lead to increased IGF2 availability. These changes in circulating IGF2 and IGFBP2 in response to LFD are sex-dependent and could be involved in the differential impact of poor dietary habits on the homeostatic circuitry regulating metabolism (37, 66).

In the hypothalamus we found no effect of short-term dietary challenge on the mRNA levels of IGF1. In contrast, both IGF2 and IGFBP2 mRNA levels were increased in males after LFD intake. The metabolic effects of IGF2 and IGFBP2 in the brain are largely unknown, but our results suggest that these factors may also participate in metabolic control in the hypothalamus. The fact that the LFD had more effects on the IGF system than did the HFD, at least during short-term intake, raises two important considerations. First, studies where a LFD is used as a control diet for HFD intake do not necessarily reflect changes in response to high fat intake, but could reflect what is occurring in response to the LFD. Thus, comparison to a normal chow diet is important. Secondly, the question becomes why does the LFD induce these changes? Although the LFD and chow diet used here have similar percentages of carbohydrates, proteins and fats, the carbohydrate composition is quite different. The amount of sucrose is considerably higher (33.1%) in the LFD compared to the chow (0.9%) or HFD (8.9%). Thus, the possibility that the changes in IGF2 and IGFBP2 are related to specific nutrients, such as sucrose, deserves further investigation. Previous studies indicate sex specific metabolic responses to sucrose intake (67) and even though no effect was observed on body weight in either sex after 2 weeks of a high-sucrose diet, Busserolles and colleagues found that females were more resistant to the pro-oxidant effects of this diet (68).

Excess HFD consumption can lead to important changes in the brain, promoting gliosis and inflammatory responses (69, 70). This involves astrocyte and microglia activation, which is initially protective (71, 72) but when prolonged can become damaging (73), leading to neuronal death in the arcuate nucleus (74). There was an overall effect of diet on hypothalamic GFAP levels, with LFD inducing a slight increase in both males and females, but no changes in the levels of the microglial marker Iba1 or activation of inflammatory pathways were found in response to either diet. Although HFD is reported to induce hypothalamic gliosis/inflammation in less than a week, which then wains only to reappear a couple of weeks later (70). Other studies report that at 1 week of HFD intake no signs of hypothalamic gliosis/inflammation can be detected (75, 76), similar to that observed here. It is possible that the initial protective glial/inflammatory reaction to excess fat intake begins to switch after ~1 week of continuous exposure to this toxic diet, transitioning from a protective to a harmful response. This hypothesis obviously needs further investigation.

The mRNA levels of NPY and AgRP increased after LFD consumption, but only in males and with no effect of HFD. There were no changes in POMC mRNA levels in response to either diet. Insulin suppresses the hypothalamic orexigenic circuitry to reduce food intake (77) and has been shown to decrease both NPY and AgRP mRNA levels in hypothalamic cells *in vitro* (78). As IGF1 and IGF2 have “insulin-like” effects it is possible that they are involved in the modulation of metabolic neuropeptides. Indeed, IGF1 has been shown to modulate POMC mRNA levels (31), but whether IGF2 has metabolic effects at the hypothalamic levels remains unknown. It is possible that the higher expression of both orexigenic neuropeptides after LFD consumption in males is due to the higher sucrose content of this diet. We previously reported that normal male Wistar rats given a 33% sucrose solution instead of water for 2 months had increased NPY and AgRP mRNA levels, with no changes in POMC or CART mRNA expression (79).

One of the novel observations reported here is that there are sex specific changes in IGF2 and IGFBP2, both systemically and centrally, in response to short-term dietary changes. Sex differences in the response to metabolic challenges and the propensity to become obese, as well as to develop complications associated with obesity, have been widely reported (37, 66, 80). The metabolic responses to manipulations of both IGF2 and IGFBP2 have also been shown to be different between males and females. For example, in IGFBP2 KOs males becoming overweight in adulthood while this does not occur in females, at least in young adults (81). Hypothalamic IGF2 expression is upregulated in the female offspring of mothers ingesting a HFD, while this is not observed in males (82). Both IGF2 and IGFBP2 expression levels are modified by estrogens in various tissues including the brain (83–85), suggesting that the sex steroid environment participates in the control of these two factors. Moreover, there is a clear interaction between estrogens and the IGF system (86) and these two hormonal systems are sexually dimorphic, as well as their interactions. The physiological meaning or outcome of these sex differences in the IGF system and their effects on metabolism have yet to be determined, but it is clear that studies aimed to understand metabolic disarray and in the search for treatments of obesity must take this into consideration.

In conclusion, short-term LFD intake induced more changes in both the central and peripheral IGF system than did HFD, with these effects being different in males and females. As bodyweight was not changed in response to either diet, it is possible that the observed changes in the IGF system are related to the dietary composition. This possibility deserves further investigation.

## Data Availability Statement

The raw data supporting the conclusions of this article will be made available by the authors, without undue reservation.

## Ethics Statement

All experiments were designed according to the European Communities Council Directive (2010/63/UE) and the Royal Decree 53/2013 pertaining to the protection of experimental animals. This study was also approved by the Ethical Committee of Animal Experimentation of the Hospital Puerta de Hierro de Madrid and the Animal Welfare Organ of the Comunidad Autónoma de Madrid.

## Author Contributions

JA and JC: conception and design of study. SG-C, FD, PR, and AF-R: animal handling. SG-C, MJ-H, and VB: biochemical analysis. SG-C, LF, and JC: data analysis. SG-C, LF, and JC: redaction of manuscript. SG-C, LF, FD, PR, MJ-H, AF-R, VB, JA, and JC: revision of manuscript. All authors: contributed to the article and approved the submitted version.

## Conflict of Interest

The authors declare that the research was conducted in the absence of any commercial or financial relationships that could be construed as a potential conflict of interest.
